# Hif-1α–Induced Expression of Il-1β Protects against Mycobacterial Infection in Zebrafish

**DOI:** 10.4049/jimmunol.1801139

**Published:** 2018-12-14

**Authors:** Nikolay V. Ogryzko, Amy Lewis, Heather L. Wilson, Annemarie H. Meijer, Stephen A. Renshaw, Philip M. Elks

**Affiliations:** * The Bateson Centre, University of Sheffield, Western Bank, Sheffield S10 2TN, United Kingdom;; † Centre for Inflammation Research, University of Edinburgh, Edinburgh EH16 4TJ, United Kingdom;; ‡ Department of Infection and Immunity and Cardiovascular Disease, University of Sheffield, Western Bank, Sheffield S10 2RX, United Kingdom; and; § Institute of Biology, Leiden University, 2333 CC Leiden, the Netherlands

## Abstract

Drug-resistant mycobacteria are a rising problem worldwide. There is an urgent need to understand the immune response to tuberculosis to identify host targets that, if targeted therapeutically, could be used to tackle these currently untreatable infections. In this study we use an Il-1β fluorescent transgenic line to show that there is an early innate immune proinflammatory response to well-established zebrafish models of inflammation and *Mycobacterium marinum* infection. We demonstrate that host-derived hypoxia signaling, mediated by the Hif-1α transcription factor, can prime macrophages with increased levels of Il-1β in the absence of infection, upregulating neutrophil antimicrobial NO production, leading to greater protection against infection. Our data link Hif-1α to proinflammatory macrophage Il-1β transcription in vivo during early mycobacterial infection and importantly highlight a host protective mechanism, via antimicrobial NO, that decreases disease outcomes and that could be targeted therapeutically to stimulate the innate immune response to better deal with infections.

## Introduction

Pulmonary tuberculosis (TB) is a major world health problem caused by the bacillus *Mycobacterium tuberculosis* (1). It is a current priority for infectious disease research because of increasing rates of multi- and totally drug–resistant strains causing high levels of mortality, especially in the immunocompromised (2). Mycobacteria are specialized at evading killing mechanisms of the immune system to survive. Mycobacteria and immune cells create a highly organized niche, called the granuloma, in which *M. tuberculosis* can proliferate or enter a latent phase, protected from the immune system (3, 4). In human *M. tuberculosis* infection, bacteria first encounter cells of the innate immune system in and around the lungs, either macrophages in the alveolar space or neutrophils in the surrounding capillary vasculature, before the involvement of adaptive immunity and granuloma formation (5, 6). These initial phagocytosis events are followed by the attraction of other innate immune cells which signal to draining lymph nodes to activate the adaptive immune response, signs of which only become apparent 3–8 weeks postinfection in humans (6). Although granuloma formation is reasonably well characterized, the initial interactions of the bacteria with the host innate immune cells are less well defined in vivo.

*M. tuberculosis*, like many other bacterial and pathogenic microbes, triggers a proinflammatory immune response via the activation of TLRs (7). The activation of the innate immune cells via TLR signaling is a critical early host response to many invading pathogens for successful clearance of infection, and, in the absence of TLR signaling, mycobacteria grow unchecked to cause systemic infection (8). Although mycobacteria can hijack host leukocytes to create a niche for their growth, in zebrafish models, many of the initial *M. marinum* inoculum are neutralized by macrophages and neutrophils before infection can take hold (9, 10). Early mycobacterial interaction with host leukocytes is critical for the pathogen, and manipulation of the macrophage by the bacteria is required for establishment of a permissive niche in which the bacteria can grow and build its host-derived protective structure, the granuloma (11, 12). Indeed, the control of the macrophage by *M. marinum* may happen early in infection, as there is a phase of infection from 6 h to 1 d postinfection (dpi) in the zebrafish model that is characterized by a dampening of the cytokine transcriptional response (13). Greater understanding of the diverse phenotype of macrophages immediately postinfection may allow therapeutic tuning to provide maximal early control of mycobacteria during infection (14, 15). Recent studies in optically translucent zebrafish infection models have indicated that initial interactions between *M. marinum* and macrophages and neutrophils are more complex than originally thought, with successive rounds of bacterial internalization and leukocyte cell death leading to granuloma formation (9, 16, 17). The immune molecular mechanisms involved in these early processes are poorly understood.

We have previously demonstrated in a zebrafish/*M. marinum* model of TB that the initial immune response to infection can be enhanced by stabilizing host-derived hypoxia-inducible factor-1 α (Hif-1α), leading to reduced bacterial burden (18). Hif-1α is a major transcriptional regulator of the cellular response to hypoxia, which has been implicated in the activation of macrophages and neutrophils during infection and inflammatory processes (19, 20). Stabilization of Hif-1α in zebrafish upregulated proinflammatory neutrophil NO production, leading to lower mycobacterial burden (18, 21). The mechanisms by which proinflammatory cytokines associated with this NO increase are regulated by Hif-1α signaling is not known.

Il-1β is a critical macrophage-derived activator of immune cells with wide-ranging and complex effects on immune signaling and downstream pathways. Il-1β has been shown to be upregulated in the onset and formation of *M. marinum* and *M. tuberculosis* granulomas (22–24). We hypothesized that Il-1β would be activated in specific immune cell populations early in *M. marinum* infection (within 1 dpi, pregranuloma formation) and that Hif-1α acts via altered expression of this important proinflammatory mediator to confer protection against mycobacterial infection. In this study, using the zebrafish *M. marinum* model and fluorescent transgenic lines, we show that *il-1β* is transcriptionally upregulated in macrophages early during in vivo infection. Stabilization of Hif-1α upregulates *il-1β* transcription in macrophages in the absence of infection. *il-1β* signaling is required for protective NO production by neutrophils and a subsequent decrease in infection. Our data indicate that protective Hif-1α mediated NO is at least partially regulated by the key proinflammatory mediator Il-1β, increasing our understanding of the mechanism of action of the potential therapeutic target, Hif-1α, as a host-derived factor in TB.

## Materials and Methods

### Zebrafish and bacterial strains

Zebrafish were raised and maintained on a 14:10 h light/dark cycle at 28°C, according to standard protocols (25), in U.K. Home Office–approved facilities at The Bateson Centre aquaria at the University of Sheffield. Strains used were Nacre (wild type), *Tg(mpeg1:mCherry-F)ump2Tg, TgBAC(il-1β:eGFP)sh445*, *Tg(mpeg1:mCherryCAAX)sh378*, *Tg(phd3:EGFP)i144*, and *Tg(lyz:Ds-RED2)nz50* (26–30).

*M. marinum* infection experiments were performed using *M. marinum* M (no. BAA-535; ATCC) containing a psMT3-mCherry or psMT3 mCrimson vector (31). Injection inoculum was prepared from an overnight liquid culture in the log-phase of growth resuspended in 2% polyvinylpyrrolidone (PVP) 40 solution (Calbiochem) as previously described (32). One hundred to one hundred and fifty CFU were injected into the caudal vein at 28–30 h postfertilization (hpf) as previously described (33).

### Generation of *TgBAC(il-1β:GFP)sh445* transgenic and *il-1β^SH446^/il-1β^SH446^* mutant zebrafish

An eGFP SV40 polyadenylation cassette was inserted at the *il-1β* ATG start site of the zebrafish bacterial artificial chromosome (BAC) CH-211-147h23 using established protocols (34). Inverted Tol2 elements were inserted into the chloramphenicol coding sequence, and the resulting modified BAC was used to generate *TgBAC(il-1β:eGFP)sh445*.

*il-1*^−/−^ (*il-1β^SH446^/il-1β^SH446^*) mutant embryos were generated by CRISPR–Cas9 mediated mutagenesis targeted around an Mwo1 restriction site in the third exon of *il-1β* using the method described by Hruscha et al. (35) and the template sequence 5′-AAAGCACCGACTCGGTGCCACTTTTTCAAGTTGATAACGGACTAGCCTTATTTTAACTTGCTATTTCTAGCTCTAAAAC**TGAGCATGTCCAGCACCTC**GGCTATAGTGAGTCGTATTACGC-3′ (*il-1β* target sequence in bold). PCR with *il-1gF* 5′-TAAGGAAAAACTCACTTC-3′ and *il-1gF* 5′-ATACGTGGACATGCTGAA-3′ and subsequent Mwo1 digestion were used for genotyping.

### Morpholino knockdown of *il-1β*

The *il-1β* morpholino (Gene Tools) was used as previously reported (36). A standard control morpholino (Gene Tools) was used as a negative control. RT-PCR of *il-1β* was performed on embryos at 2 and 5 d postfertilization (dpf), as previously described (36). The following primers were used: *il-1β*, accession number NM_212844 (https://www.ncbi.nlm.nih.gov/nuccore/NM_212844.2), forward primer: 5′-ATGGCATGCGGGCAATATGAA-3′, reverse primer: 5′-CACTTCACGCTCTTGGATGA-3′; *ppia1* control, accession number AY391451 (https://www.ncbi.nlm.nih.gov/nuccore/AY391451), forward primer: 5′-ACACTGAAACACGGAGGCAAG-3′, reverse primer: 5′-CATCCACAACCTTCCCGAACAC-3′.

### Confocal microscopy of transgenic larvae

1 and 4 dpi, transgenic zebrafish larvae infected with fluorescent *M. marinum* strains were mounted in 0.8–1% low melting point agarose (Sigma-Aldrich) and imaged on a Leica TCS SPE confocal on an inverted Leica DMi8 base and imaged using 20× or 40× objective lenses.

For quantification purposes, acquisition settings and area of imaging (in the caudal vein region) were kept the same across groups. Corrected total cell fluorescence was calculated for each immune-stained cell using Image J as previously described (18, 21).

### Tailfin transection

Inflammation was induced in zebrafish embryos by tail transection at 2 or 3 dpf as described previously (34). Embryos were anesthetized by immersion in 0.168 mg/ml Tricaine (Sigma-Aldrich), and tail transection was performed using a microscalpel (World Precision Instruments).

### Quantitative PCR of *il-1β*

SYBR Green quantitative PCR (qPCR) was performed on 1dpi *M. marinum* infected (or PVP control) embryos as previously described (37). The following primers were used: *il-1β*, accession number NM_212844 (https://www.ncbi.nlm.nih.gov/nuccore/NM_212844.2), forward primer: 5′-GAACAGAATGAAGCACATCAAACC-3′, reverse primer: 5′-ACGGCACTGAATCCACCAC-3′; *ppia1* control, accession number AY391451 (https://www.ncbi.nlm.nih.gov/nuccore/AY391451), forward primer: 5′-ACACTGAAACACGGAGGCAAG-3′, reverse primer: 5′-CATCCACAACCTTCCCGAACAC-3′.

### Bacterial pixel count

*M. marinum* mCherry–infected zebrafish larvae were imaged at 4 dpi on an inverted Leica DMi8 with a 2.5× objective lens. Brightfield and fluorescent images were captured using a Hamamatsu OrcaV4 camera. Bacterial burden was assessed using dedicated pixel counting software as previously described (38).

### RNA injections

Embryos were injected with dominant *hif-1αb* variant RNA at the one-cell stage as previously described (20). *hif-1α* variants used were dominant active (DA) and dominant negative (DN) *hif-1α* (ZFIN: *hif1ab*). Phenol red (PR) (Sigma-Aldrich) was used as a vehicle control.

### Hydroxylase inhibitors

Embryos were treated from 32 hpf until 2 dpf by addition to the embryo water, and DMSO was used as a negative solvent control. The pan hydroxylase inhibitor, dimethyloxaloylglycine (DMOG; Enzo Life Sciences), was used at a 100-μM concentration by incubation in E3 embryo media as previously described (20). The selective PHD inhibitor JNJ-402041935 (Cayman Chemicals) was used at 100 μM (39).

### Hypoxia incubation of embryos

Embryos were incubated in 5% oxygen (with 5% carbon dioxide) in a hypoxia hood (SCI-tive UM-027; Baker Ruskinn) from 32 h postinfection for 6 or 16 h and were imaged at 2 dpf. Embryos from the same clutch kept in incubated normoxic room air were used as controls.

### Anti-nitrotyrosine Ab staining

Larvae were fixed in 4% paraformaldehyde in PBS overnight at 4°C, and nitrotyrosine levels were immune labeled using a rabbit polyclonal anti-nitrotyrosine Ab (06-284; Merck Millipore) and were detected using an Alexa Fluor–conjugated secondary Ab (Invitrogen Life Technologies) as previously described (18, 21).

### Statistical analysis

All data were analyzed (Prism 7.0, GraphPad Software) using unpaired, two-tailed *t* tests for comparisons between two groups and one-way ANOVA (with Bonferroni posttest adjustment) for other data. The *p* values shown are *p < 0.05, **p < 0.01, and ***p < 0.001.

## Results

### *il-1β:GFP* is upregulated in macrophages during early and later stage *M. marinum* infection

**FIGURE 1.**
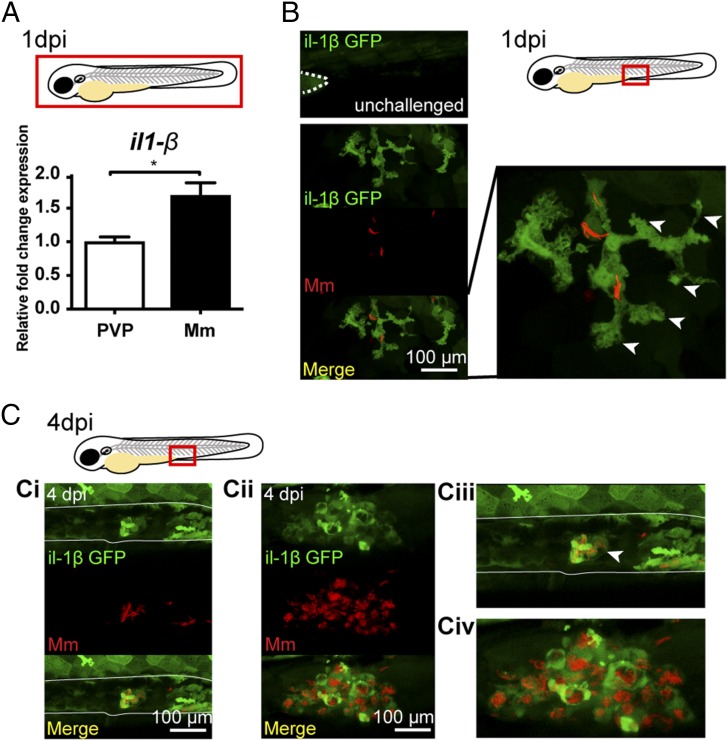
*TgBAC(il-1β:GFP)sh445* is upregulated by *M. marinum* (Mm) in infected macrophages at early and later stage infection. (**A**) Graph showing relative whole-body *il-1β* mRNA expression in whole embryos by SYBR Green qPCR in *M. marinum* infected 1 dpi larvae and mock-injected (PVP) controls. Data shown are mean ± SEM (*n* = 3 independent experiments). *p < 0.05. (**B**) Fluorescent confocal micrographs of 1 dpi larvae, prior to granuloma formation. Unchallenged *TgBAC(il-1β:GFP)sh445* has no detectable expression in immune cells and low detectable levels in the yolk (dotted line) and some muscle cells. *il-1β* expression was detected by GFP levels, in green, using the *TgBAC(il-1:eGFP)sh445* transgenic line. *M. marinum* mCherry is shown in the red channel. Increased levels of *il-1β:GFP* expression were detectable in cells associated with infection. Infected macrophages with *il-1β-GFP* levels have an activated, branched phenotype (white arrowheads). (**C**) Fluorescent confocal micrographs of 4-dpi larvae. *il-1β* expression was detected by GFP levels, in green, using the *TgBAC(il-1:eGFP)sh445* transgenic line. *M. marinum* mCherry is shown in the red channel. Increased levels of *il-1β:GFP* expression were detectable in immune cells that are in the blood vessels [(C**i**) and blown up in (C**iii**), blood vessel indicated by solid white lines] and in early tissue granulomas [(C**ii**) and blown up in (C**iv**)].

Early *M. marinum* infection in zebrafish is characterized by a period of increased proinflammatory signaling (9, 13). Levels of proinflammatory cytokines have only been previously studied at a transcriptional level in whole embryos or FACS-sorted cells rather than detecting levels in situ, over time, in an intact organism (13). We hypothesized that Il-1β is a major proinflammatory cytokine that would be upregulated by both mycobacterial infection and Hif-1α stabilization. We have previously shown upregulation of *il-1β* message after induction of inflammation via tailfin transection by qPCR and wholemount in situ hybridization (WISH) in the zebrafish (40). *il-1β* is one of the most readily detectable proinflammatory cytokines during early granuloma stages of *M. marinum* infection and at 1 dpi (Fig. 1A) (37). At 1 dpi, transcription is upregulated 1.7-fold measured by qPCR, compared with PVP injection controls (Fig. 1A). Macrophage expression of *il-1β* is greatly underrepresented when measured in this way on the whole-body level because of the small proportion of cells that contribute to the immune lineage. Therefore, to investigate *il-1β* expression on a cellular level in vivo, we developed a BAC-derived *il-1β* promoter–driven GFP line, *TgBAC(il-1β:GFP)SH445*, to assess *il-1β* expression in real time during mycobacterial infection. We sought to examine *il-1β:GFP* expression in our well-established inflammation assay before investigating its expression during mycobacterial infection. WISH of *il-1β* and *il-1β:GFP* does not exhibit any immune cell expression under basal conditions (Supplemental Fig. 1A, 1B, Fig. 1B). *il-1β:GFP* recapitulates *il-1β* WISH expression in response to tail transection, with upregulation observed in cells in and around the caudal hematopoietic region, consistent with immune cell expression (Supplemental Fig. 1A, 1B) (40), although, as expected, the synthesis of GFP occurs over a longer timescale than that of *il-1β* mRNA detected by WISH. Neutrophils are the first cells to respond to tailfin transection with increased *il-1β:GFP*, with fluorescence first observed at 1 h postwounding (hpw) and still present at 6 hpw (Supplemental Fig. 1C). Having demonstrated that the *il-1β:GFP* is responsive to inflammation in similar cells over a similar timespan as the in situ hybridization, we sought to investigate its regulation during mycobacterial infection.

**FIGURE 2.**
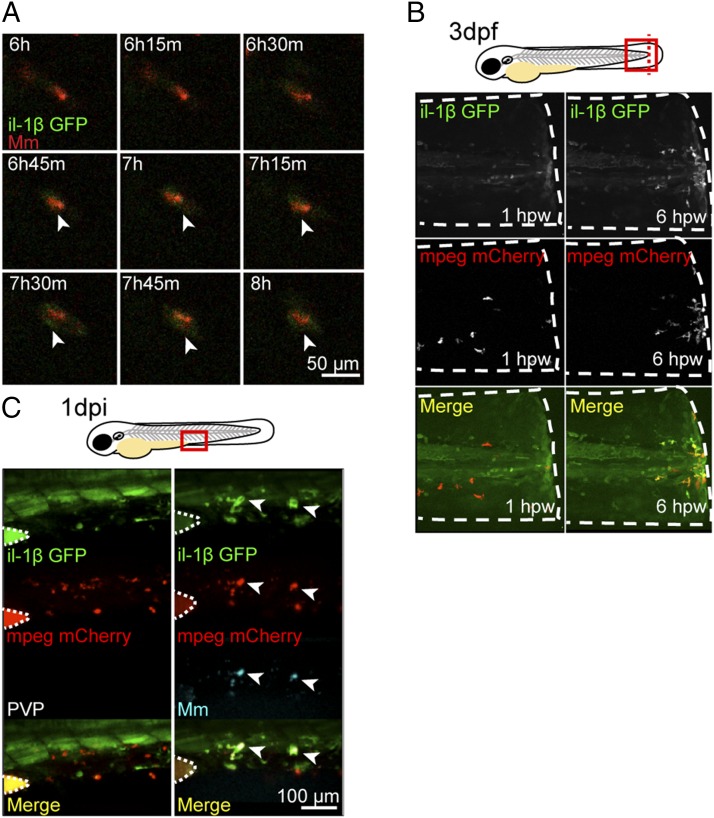
*il-1β:GFP* is activated 6–8 h after challenge in macrophages. (**A**) Fluorescent confocal micrographs of a time lapse between 6 and 8 h post *M. marinum* infection. *M. marinum* mCherry is shown in the red channel and *il-1β:GFP* is shown in the green channel, with the microscope settings set to detect low GFP levels. Arrowheads indicate the emergence of *il-1β:GFP* expression in an infected cell. (**B**) Fluorescent confocal micrographs of *TgBAC(il-1β:GFP)sh445* crossed to *Tg(mpeg1:mCherryCAAX)sh378* line labeling macrophages. The tailfin was transected at 3 dpf, and fluorescence imaging was performed at the wound at 1 hpw and 6 hpw. Red macrophages are not positive for *il-1β:GFP* expression at 1 hpw, and the first detectable *il-1β:GFP* expression is found in the macrophages at 6 hpw. (**C**) Fluorescent confocal micrographs of 1 dpi caudal vein region of infection. *il-1β* expression was detected by GFP levels, in green, using the *TgBAC(il-1β:eGFP)sh445* transgenic line. Macrophages are shown in red using a *Tg(mpeg1:mCherryCAAX)sh378* line. *M. marinum* Crimson is shown in the blue channel (right panels) with a PVP control (left panels). Without infection there is little overlap of *il-1β:GFP* and *mpeg:mCherry*, whereas in infected larvae macrophages have higher levels of *il-1β:GFP*. Arrowheads indicate infected macrophages with high levels of *il-1β:GFP.* Dotted lines indicate the yolk extension of the larvae where there is nonspecific fluorescence.

We used the *TgBAC(il-1β:GFP)sh445* line to show that GFP is expressed in cells proximal to *M. marinum* infection sites at pregranuloma phases (1 dpi) (Fig. 1B) and in larval granulomas (4 dpi) (Fig. 1C). Many of these cells contained *M. marinum* and had the appearance of activated immune cells with a dynamic branched phenotype (Fig. 1B, Supplemental Video 1). The earliest timepoint at which *il-1β:GFP* could be detected by confocal microscopy was between 6 and 8 h postinfection, (Fig. 2A), consistent with rapid transcriptional activation of the *il-1β* promoter postinfection and similar to the timing of macrophage *il-1β:GFP* expression after tailfin transection (Fig. 2B). *il-1β:GFP* was predominantly upregulated in infected macrophages at 1 dpi (Fig. 2C), consistent with their containment of phagocytosed *M. marinum* (Fig. 1B). These data demonstrate that during early stages of infection, *il-1β* is transcriptionally activated in infected macrophages as part of an early proinflammatory response.

### Stabilization of Hif-1α upregulates *il-1β:GFP* at early stages of infection

**FIGURE 3.**
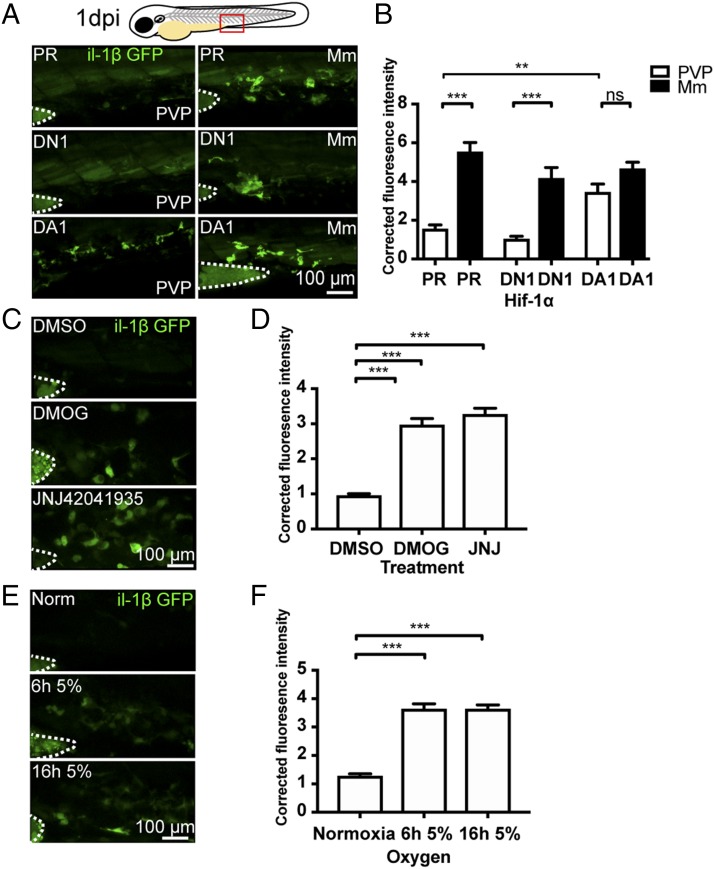
*il-1β:GFP* is upregulated in the absence of infection by stabilized Hif-1α. (**A**) Fluorescent confocal micrographs of 1 dpi caudal vein region of infection. *il-1β:GFP* expression was detected by GFP levels, in green, using the *TgBAC(il-1β:eGFP)sh445* transgenic line. Larvae were injected at the one-cell stage with DN or DA Hif-1α or PR control. Noninfected larvae are in the left panels (PVP), and *M. marinum* Crimson infected larvae are in the right panels (*M. marinum*). Dotted lines indicate the yolk extension of the larvae where there is nonspecific fluorescence. (**B**) Corrected fluorescence intensity levels of *il-1β:GFP* confocal z-stacks in uninfected larvae (PVP, empty bars) and infected larvae (*M. marinum*, filled bars) at 1 dpi. DA Hif-1α (DA1) had significantly increased *il-1β:GFP* levels in the absence of *M. marinum* bacterial challenge compared with PR and DN Hif-1α (DN1)–injected controls. Data shown are mean ± SEM (*n* = 24–48 cells from four to eight embryos representative of three independent experiments). (**C**) Fluorescent confocal micrographs of 2 dpf caudal vein region in the absence of infection. *il-1β:GFP* expression was detected by GFP levels, in green, using the *TgBAC(il-1β:eGFP)sh445* transgenic line. Larvae were treated with hypoxia mimetics (hydroxylase inhibitors) DMOG and JNJ42041935 or solvent control (DMSO). Dotted lines indicate the yolk extension of the larvae where there is nonspecific fluorescence. (**D**) Corrected fluorescence intensity levels of *il-1β:GFP* confocal z-stacks in uninfected larvae at 2 dpf after treatment with DMOG and JNJ42041935 or solvent control (DMSO). Data shown are mean ± SEM (*n* = 108 cells from 18 embryos accumulated from three independent experiments). (**E**) Fluorescent confocal micrographs of 2 dpf caudal vein region in the absence of infection. *il-1β:GFP* expression was detected by GFP levels, in green, using the *TgBAC(il-1β:eGFP)sh445* transgenic line. Larvae were raised in 5% oxygen (hypoxia) for 6 h (6 h 5%) or 16 h (16 h 5%) at 32 hpf and imaged at 48 hpf. Dotted lines indicate the yolk extension of the larvae where there is nonspecific fluorescence. (**F**) Corrected fluorescence intensity levels of *il-1β:GFP* confocal z-stacks in uninfected larvae at 2dpf after treatment with DMOG and JNJ42041935 or solvent control (DMSO). Data shown are mean ± SEM (*n* = 108 cells from 18 embryos accumulated from three independent experiments). **p < 0.01, ***p < 0.001.

We have previously shown that stabilization of Hif-1α induces neutrophil proinflammatory NO production (18, 21). We hypothesized that this may be a part of an increased proinflammatory profile in innate immune cells; therefore, we tested whether Hif-1α is upregulating a proinflammatory program in the absence of infection using the *il-1β:GFP* transgenic line. DA Hif-1α significantly increased *il-1β:GFP* expression in the absence of *M. marinum* infection at 2 dpf, whereas DN Hif-1α caused no difference in *il-1β:GFP* expression (Fig. 3A, 3B).

To assess whether stabilization of physiological levels of Hif-1α is sufficient to induce *il-1β:GFP* expression in the absence of *M. marinum* infection, embryos were treated with the hydroxylase inhibitors DMOG and JNJ-402041935 (20, 39). Hydroxylase inhibitors stabilize endogenously produced levels of Hif-1α by blocking hydroxylation by the PHD and FIH hydroxylase enzymes. Both hydroxylase inhibitors increased *il-1β:GFP* in the absence of infection (Fig. 3C, 3D) to a similar extent as that observed with DA Hif-1α (Fig. 3A, 3B). Finally, to understand whether a physiological stimulus could also induce *il-1β:GFP* expression, zebrafish were incubated in physiological hypoxia. The lowest level of oxygen zebrafish embryos tolerate without developing abnormally is 5% oxygen (and 5% carbon dioxide) (26). To demonstrate that this level of hypoxia activated Hif-1α, two time periods were tested in the *Tg(phd3:EGFP)i144* transgenic line. We have previously demonstrated that *phd3* is a major downstream target of hypoxia in zebrafish embryos and that this transgenic line accurately reports Hif-1α activation (26). Incubation in 5% oxygen overnight (for 16 h) mimicked the time period used for the hydroxylase inhibitors and robustly activated *phd3:GFP* (Supplemental Fig. 2A, 2C); however, due to the effects of limiting oxygen on other pathways (including metabolomic pathways), the zebrafish at 2 dpf were developmentally delayed (denoted by smaller eyes and less pigment). Five percent oxygen incubation for a shorter 6-h period was sufficient to activate *phd3:GFP* by 2 dpf without any overt developmental delay (Supplemental Fig. 2A, 2B). Both time periods of 5% oxygen incubation were sufficient to increase levels of *il-1β:GFP* in the absence of infection to a similar extent to both hydroxylase inhibition and DA Hif-1α (Fig. 3E, 3F).

Together, these data indicate that *il-1β* expression is part of a proinflammatory response to increased Hif-1α levels that could aid the host response to *M. marinum* challenge.

### Inhibition of *il-1β* increases *M. marinum* burden and inhibits the Hif-1α NO response

**FIGURE 4.**
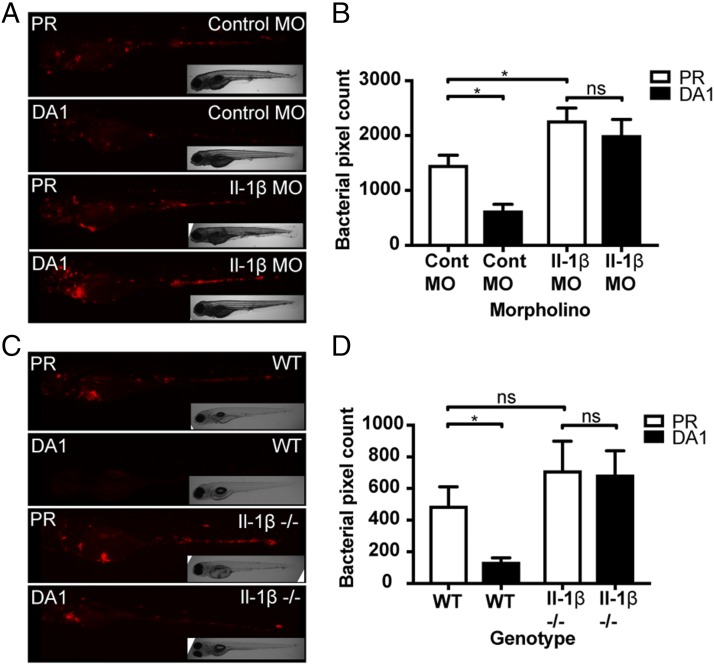
*il-1β* knockdown abrogates the protective effect of DA Hif-1α on bacterial burden. (**A**) Stereo-fluorescence micrographs of *M. marinum* mCherry infected 4 dpi larvae after injection with DA Hif-1α (DA1) and the *il-1β* morpholino (Il-1β MO), using the standard control morpholino and PR (Control) as a negative control. (**B**) Bacterial burden of larvae shown in (A). Data shown are mean ± SEM (*n* = 46–50 as accumulated from three independent experiments). (**C**) Stereo-fluorescence micrographs of *M. marinum* mCherry infected 4 dpi larvae after injection with DA Hif-1a (DA1) or PR (negative control) in an *il-1β* mutant (−/−) and WT (sibling +/+) background. (**D**) Bacterial burden of larvae shown in (C). Data shown are mean ± SEM (*n* = 16–20 as accumulated from three independent experiments). *p < 0.05.

IL-1β is a major proinflammatory cytokine that in many infections is instrumental in coordinating the immune response (41, 42). We sought to test whether Il-1β was important in early *M. marinum* infection. When functional Il-1β was blocked using a well-characterized and validated *il-1β* morpholino (Supplemental Fig. 3A), the morphants showed significantly increased infection compared with control morphants (Fig. 4A, 4B).

We have previously shown that stabilization of Hif-1α induces proinflammatory neutrophil NO production, via inducible NO synthase (iNOS) (18, 21). DA Hif-1α was not sufficient to reduce *M. marinum* infection levels when *il-1β* expression was blocked (Fig. 4A, 4B), suggesting that the *il-1β* response to *M. marinum* infection is critical to control infection. These results were supported by generation of an *il-1β* null mutant (*il-1β^SH446^/il-1β^SH446^*) (Supplemental Fig. 3B–E) in which DA Hif-1α also did not decrease infection, whereas in wild type siblings, infection was reduced (Fig. 4C, 4D).

**FIGURE 5.**
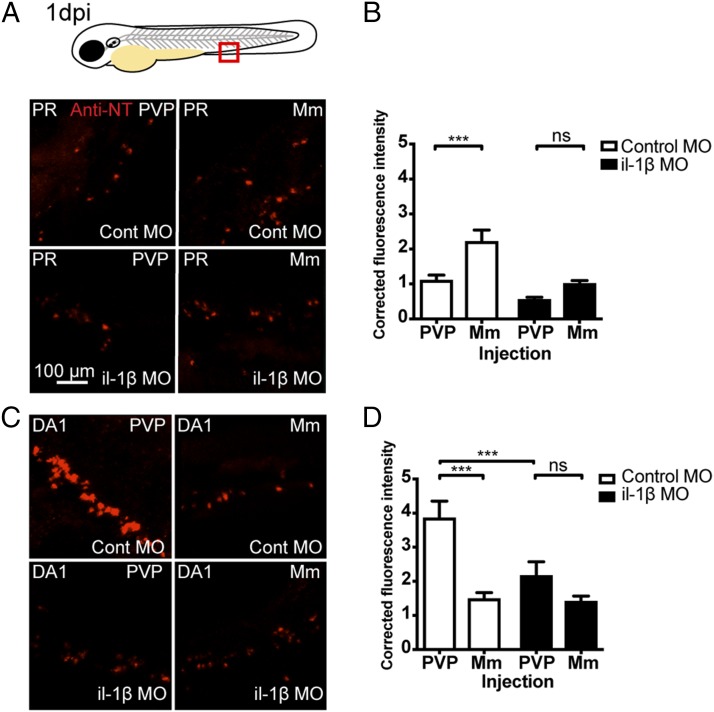
*il-1β* knockdown abrogates DA Hif-1α–dependent nitrotyrosine production. (**A**) Example fluorescence confocal z-stacks of the caudal vein region of embryos stained with Alexa Fluor 633–labeled anti-nitrotyrosine Ab (red), imaged at 1 dpi in the presence or absence of *M. marinum* infection. One-cell stage embryos were injected with PR. One-cell stage embryos we coinjected with *il-1β* morpholino or (il-1β MO) or standard control morpholino (Cont MO). At 1 dpi, larvae were infected with either *M. marinum* mCherry (*M. marinum*) or PVP as a noninfected control (*M. marinum* channel not shown in these panels). (**B**) Example fluorescence confocal z-stacks of the caudal vein region of embryos stained with Alexa Fluor 633–labeled anti-nitrotyrosine Ab (red), imaged at 1 dpi in the presence or absence of *M. marinum* infection. One-cell stage embryos were injected with DA Hif-1α. One-cell stage embryos we coinjected with *il-1β* morpholino (il-1β MO) or standard control morpholino (Cont MO). At 1 dpi, larvae were infected with either *M. marinum* mCherry (*M. marinum*) or PVP as a noninfected control (*M. marinum* channel not shown in these panels). (**C**) Corrected fluorescence intensity levels of anti-nitrotyrosine Ab confocal z-stacks of PR control injected embryos in the presence or absence of *M. marinum* infection at 1 dpi. Control morpholino is shown in the clear bars and *il-1β* morpholino (il-1β MO) in the filled bars. Data shown are mean ± SEM (*n* = 54–59 cells from 10 to 12 embryos accumulated from three independent experiments). (**D**) Corrected fluorescence intensity levels of anti-nitrotyrosine Ab confocal z-stacks of DA Hif-1α (DA1) injected embryos in the presence or absence of *M. marinum* infection at 1dpi. Control morpholino is shown in the clear bars, and il-1β morpholino (il-1β MO) is shown in the filled bars. Data shown are mean ± SEM (*n* = 54–59 cells from 10 to 12 embryos accumulated from three independent experiments). ***p < 0.001.

**FIGURE 6.**
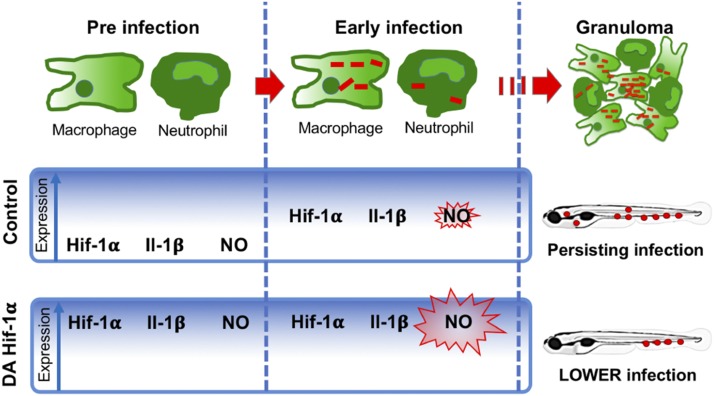
Hif-1α stabilization leads to upregulation of *il-1β* and increased neutrophil NO production that is protective against infection. During normal (control) *M. marinum* infection, Hif-1α, Il-1β, and NO transcript levels rise postinfection but are not sufficient to control infection (18). When Hif-1α is stabilized, Il-1β and subsequent neutrophil NO upregulation occurs in the absence of infection, priming the immune response to better deal with infection leading to lower burden.

NO production is found primarily in neutrophils after *M. marinum* infection in zebrafish larvae (Supplemental Fig. 4) (18, 21). We have previously demonstrated that inhibiting production of NO by Nos2 can block the antimicrobial effect of DA Hif-1α (18). Blocking Il-1β production also significantly dampened the neutrophil NO response after *M. marinum* infection at 1 dpi (Fig. 5A, 5B). As we have previously observed, DA Hif-1α upregulated NO in the absence of infection (PVP), an effect that is dampened by introduction of the bacteria (*M. marinum*) through currently unknown mechanisms, (Fig. 5C, 5D) (18). In this study, we find that *il-1β* morpholino blocked the increased production of nitrotyrosine by DA Hif-1α in the absence of bacteria (PVP) (Fig. 5C, 5D). These results show that Hif-1α activation of Nos2 may, at least in part, be acting through *il-1β* activation (Fig. 6) and hint at a much more complex regulation of proinflammatory signaling by Hif-1α than simply acting on Hif-responsive elements in the promoter of Nos2.

## Discussion

Antimicrobial resistance is a rising problem in TB infections worldwide, and there is an urgent need to understand the regulation of host immunity by TB so that we can target host-derived factors to help tackle disease. Our data identify an early proinflammatory response, involving macrophage *il-1β* expression, that is important for the onset of early disease but ultimately fails to control infection leading to granuloma formation. Using a well-established zebrafish *M. marinum* model of TB, we show that manipulation of Hif-1α can stimulate this proinflammatory network, aiding the host fight against infection and moving toward early clearance of infection. Specifically, we identify that Hif-1α–driven Il-1β contributes to the NO response, a response we have previously shown to be host protective (18, 21).

In this study, we took advantage of a novel transgenic zebrafish line to understand the dynamics and cell specificity of *il-1β* production in inflammation and mycobacterial infection, with a focus on the understudied early stages (<1dpi) of the innate immune response to TB infection. We confirmed that the *il-1β:GFP* expression of our line was faithful to *il-1β* transcription by following its expression in a well-characterized tailfin transection model of inflammation and comparison with in situ hybridization data (40, 34). Furthermore, the expression pattern of our BAC transgenic line closely matches another recently published BAC promoter–driven *il-1β* transgenic (43). The *il-1β:GFP* line also displayed some GFP signal in muscle and epithelial cells in the tail. Similar GFP expression can be seen when driven by NF-κB response elements (44) but not by WISH, suggesting that this might be off-target expression resulting from the promoter region missing some negative regulatory elements; however, it could also be specific expression that is at too low a level to be detectable by in situ hybridization. Although previous studies have shown *il-1β:GFP* to be upregulated in leukocytes at a tailfin transection (43), we combined the *il-1β:GFP* line with leukocyte-specific transgenics to show that neutrophils are the first to respond at the wound, with macrophages both migrating to and upregulating *il-1β:GFP* at later timepoints.

The *M. tuberculosis* granuloma is widely studied, both in terms of immunohistochemistry of human granulomas and in mammalian models (45–47). These studies have demonstrated that the granuloma is rich in proinflammatory cytokine production and can have necrotic centers that may be hypoxic. This proinflammatory environment has been observed in human TB, with Il-1β found to be in high levels in pleural fluid from TB patients with granulomas present (48). In our study, we observe that the proinflammatory response is present at pregranuloma stages. Lack of a proinflammatory response has been linked to poor treatment outcomes, indicating that this host response is important even in the presence of antimycobacterial agents (49). The upregulation of proinflammatory cytokines in mycobacterial infection has also been shown in the zebrafish/*M. marinum* larval model of TB granulomas, but previous studies have mainly relied on immunohistochemistry and/or transcriptomics data from either whole-body larvae or FACS-sorted immune cell populations (13, 27). Using live cell imaging, we found that *il-1β* transcription was upregulated at the granuloma formation stage; however, we also demonstrated that it is upregulated before the granuloma stage within 6–8 h postinfection. Upon infection, *il-1β:GFP* expression was predominantly upregulated in infected macrophages, indicating that within the first 24 h of infection, there is a macrophage proinflammatory response. Murine and human cell studies have indicated that macrophages are able to produce Il-1β a few hours after mycobacterial challenge, indicating that an early response is also present in mammalian systems, at least on a cellular level (22, 50). Our observations are in line with our previous observation of Hif-1α signaling early postinfection (detected using a *phd3:GFP* transgenic line), which was also observed in infected macrophages at 1 dpi (18), indicating that *il-1β*, alongside Hif-1α signaling, is part of an immediate proinflammatory macrophage response. This Hif-1α activation was shown to be transient, with *M. marinum* rapidly downregulating this in a live bacteria–dependent manner. Of note, we have previously shown that suppressing this transient early Hif-1α signal does not affect the outcome of infection, and this observation was replicated in the study, indicating that this natural, early Hif-1α stabilization is not sufficient to control infection (18). As with Hif-1α, our Il-1β data indicate that *M. marinum*–triggered *il-1β* is not sufficient to control infection with subsequent widespread granuloma formation at later stages; however, if primed with high *il-1β* and NO via Hif-1α, the immune response is boosted, leading to lower infection and toward early infection clearance.

We have previously demonstrated that stabilization of Hif-1α can aid the zebrafish host to control *M. marinum* infection, at least in part by priming neutrophils with increased nitrotyrosine generated by the Nos2 enzyme (18). If the Nos2 enzyme is blocked either pharmacologically or genetically, the protective effect of Hif-1α stabilization is lost (18). In our study, we show that stabilization of Hif-1α upregulates proinflammatory macrophage *il-1β* expression in the absence of an infection challenge. If Il-1β activity is repressed then Hif-1α induced reduction in bacterial burden is abrogated, alongside the Hif-1α–dependent increase in NO production. These data show regulation of both Nos2 and Il-1β by Hif-1α and that Hif-1α–driven NO production is partially dependent on Il-1β induction. Both human NOS-2 and IL-1β have HIF-responsive elements in their promoters, and direct regulation by HIF-α signaling has been previously demonstrated in vitro (51, 52). The link between HIF-1α and IL-1β has been previously demonstrated in murine macrophages via inflammatory activation by succinate, in the absence of infection (53). In a murine model of *M. tuberculosis*, it was found that HIF-1α is critical for IFN-γ–dependent control of *M. tuberculosis* infection, but it has not previously been demonstrated that HIF-1α is important for innate defense of macrophages against *M. tuberculosis* (54). Our data do not rule out direct regulation of Nos2 by Hif-1α, as blocking Il-1β is likely to have wider spread immune effects; however, they do suggest that Nos2 is partially upregulated by Il-1β in the stabilized Hif-1α context. These observations, alongside our finding that blocking Il-1β, primarily observed in macrophages, can block Hif-1α–induced neutrophil nitrotyrosine production, indicate a close interplay between macrophages and neutrophils during early mycobacterial infection that is not yet fully understood.

Il-1β is an important proinflammatory component and is one of the cytokines that has been shown to be transcriptionally depressed during the 6 h to 1 dpi period of *M. marinum*/zebrafish pathogenesis (13). Although this depression was not detectable using the *il-1β:GFP* line, presumably owing to the early transcriptional response postinfection coupled with the stability of the GFP protein, our data indicate that increased *il-1β* transcription due to Hif-1α stabilization during this early stage of *M. marinum* infection is protective to the host. Alongside transcription, the processing of Il-1β by caspases plays a crucial role in immune cell pyroptosis mediated by the inflammasome (54). Recent findings in the *M. marinum*/zebrafish model indicate that neutrophils and macrophages can efficiently phagocytose bacteria and undergo rounds of cell death and reuptake during the initial days of infection (9). Although in this study we show a role for early proinflammatory *il-1β* transcription during *M. marinum* infection, the role of Il-1β processing and inflammasome induced pyroptosis/cell death in these early *M. marinum* immune processes remain undetermined.

In conclusion, our data demonstrate an early proinflammatory response of *M. marinum*–infected macrophages in vivo. By stabilizing Hif-1α, macrophage Il-1β can be primed in the absence of infection and is protective upon *M. marinum* infection via neutrophil NO production. Therapeutic strategies targeting these signaling mechanisms could decrease the level of initial mycobacteria in patients and act to block the development of active TB by reactivation of macrophage proinflammatory stimuli. Furthermore, our findings may have important implications in other human infectious diseases in which the pathogen is able to circumvent the proinflammatory immune response to allow its survival and proliferation. Therapies that target host-derived signaling pathways such as these would be beneficial against multidrug resistant strains and could act to shorten the currently long antibiotic therapies required to clear TB from patients.

## Supplementary Material

Data Supplement
