# Supplementary material for: Hif-1α–Induced Expression of Il-1β Protects against Mycobacterial Infection in Zebrafish
Source: J Immunol. 2018 Dec 14;202(2):494–502. doi: 10.4049/jimmunol.1801139 (PMC6321843; doi:10.4049/jimmunol.1801139)
Supplement: Data Supplement [file JI_1801139.zip › JI_1801139_Supplemental_Figures_2.pdf]

Figure S1

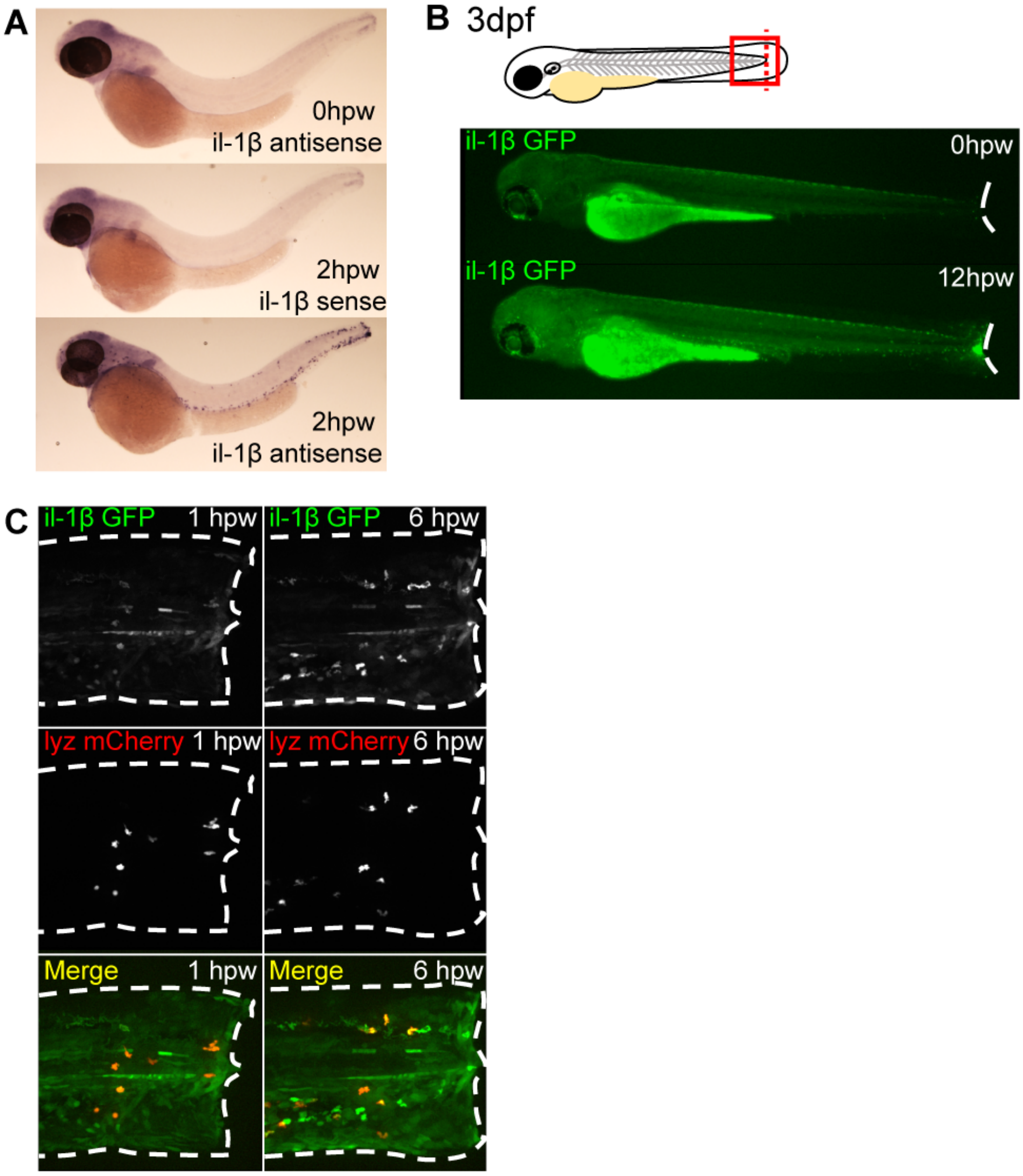

Figure S1. *TgBAC(il-1β:GFP)sh445* recapitulates *il-1β* wholemount *in situ* hybridisation pattern following sterile tailfin transection.

(A) Wholemount *in situ* hybridisation of *il-1β* in tailfin injured 2dpf embryos.

(B) Fluorescent confocal micrographs of *TgBAC(il-1β:GFP)sh445* expression after tailfin injury. Upper and lower panels show the same individual embryo 0 and 12hpi.

(C) Fluorescent confocal micrographs of *TgBAC(il-1β:GFP)sh445* crossed to *Tg(lyz:Ds- RED2)nz50* labelling neutrophils at 1 hour post wound (1hpw) and 6hpw.

Figure S2

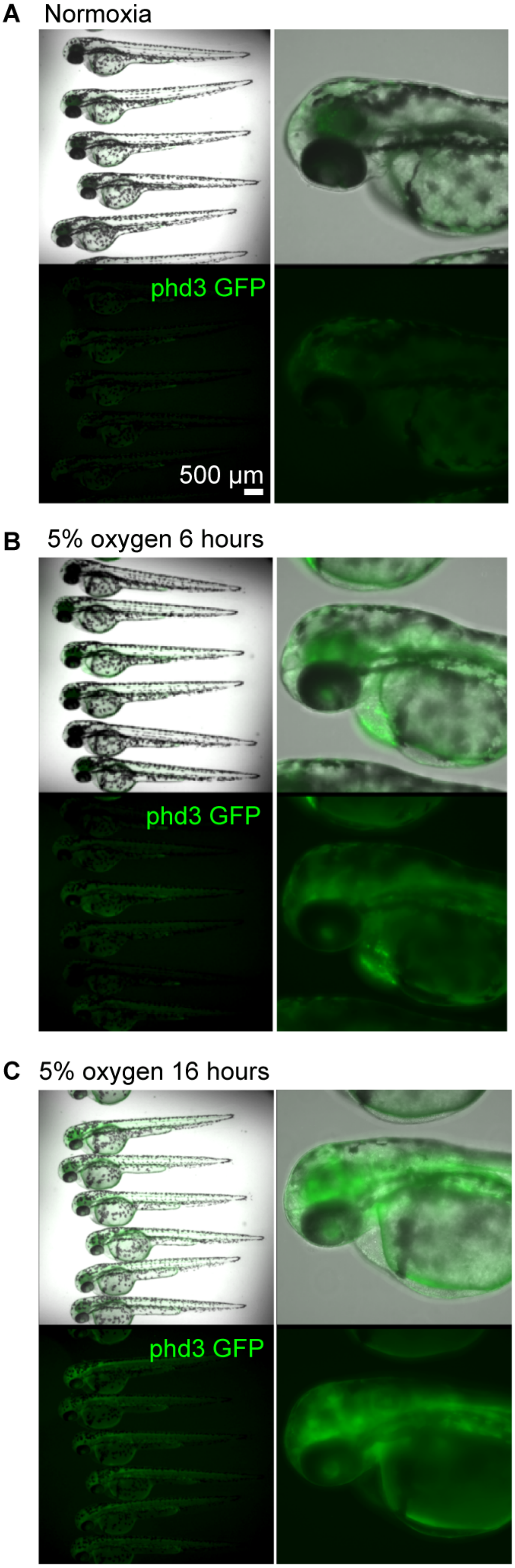

Figure S2. Exposure to hypoxia activates Hif-1α target gene expression.
(A) Brightfield and fluorescent confocal micrographs of 2dpf *Tg(phd3:EGFP)i144* after exposure to normoxia.
(B) Brightfield and fluorescent confocal micrographs of 2dpf *Tg(phd3:EGFP)i144* after exposure to 5% oxygen for 6 hours at 32hpf.
(C) Brightfield and fluorescent confocal micrographs of 2dpf *Tg(phd3:EGFP)i144* after exposure to 5% oxygen for 16 hours at 32hpf.

Figure S3

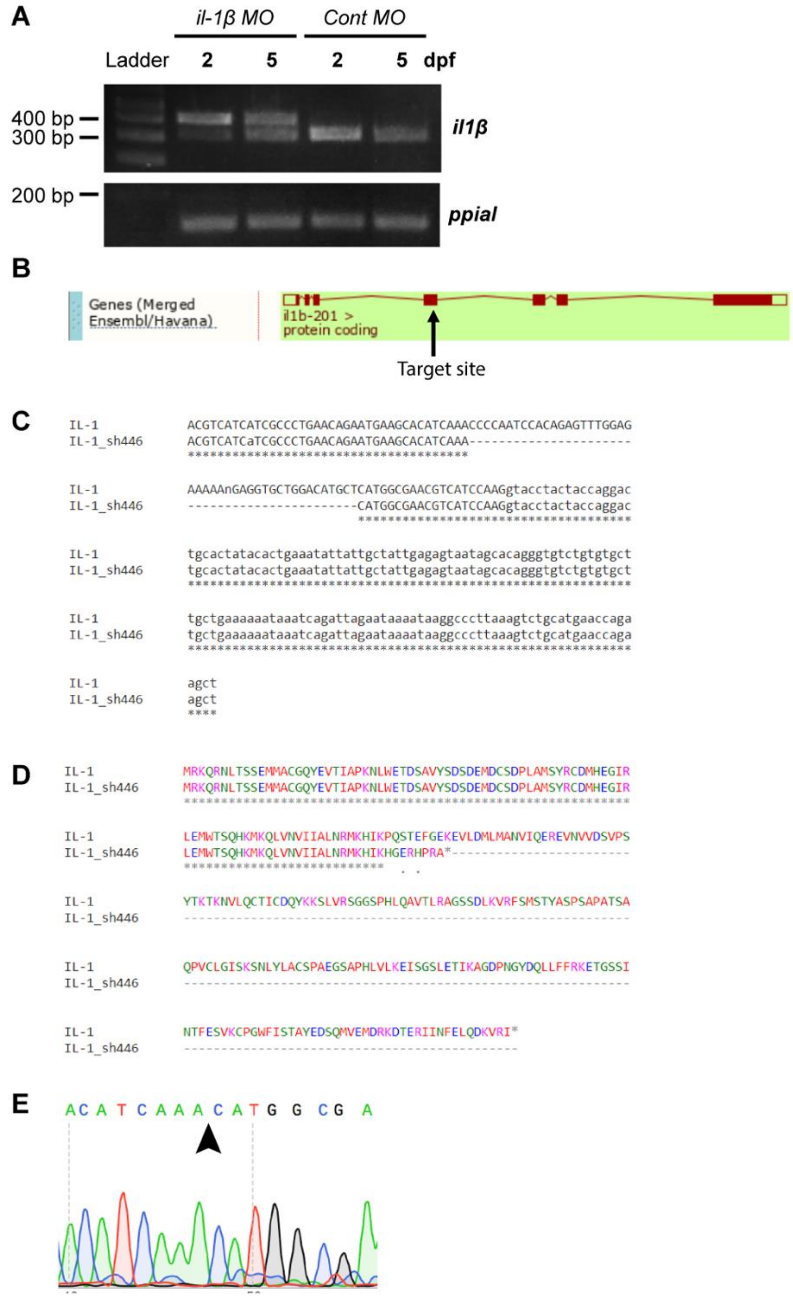

Figure S3. *il-1β* morpholino and *il-1β SH446* mutant.

(A) RT-PCR analysis demonstrating altered splicing of the *il-1β* transcript induced by the *il-1β* morpholino (*il-1β MO*) compared to control morpholino (*Control MO*) in 2dpf and 5dpf embryos. A 378bp band with an intact intron between exons 2 and 3 is present only in embryos injected with *il-1β* morpholino and is absent in controls.

(B) Screenshot of Ensembl zebrafish *il-1β* coding sequence with CRISPR-Cas9 target indicated in the fourth exon.

(C) DNA alignment of WT *il-1β* sequence and *il-1βSH446* showing the 44 base pair deletion caused by CRISPR-Cas9.

(D) Amino acid alignment of WT *il-1β* sequence and *il-1βSH446* with arrowhead showing the premature stop and removal of the putative Il-1β cleavage site.

(E) Sequencing trace showing position of CRISPR-Cas9 induced deletion.

Figure S4

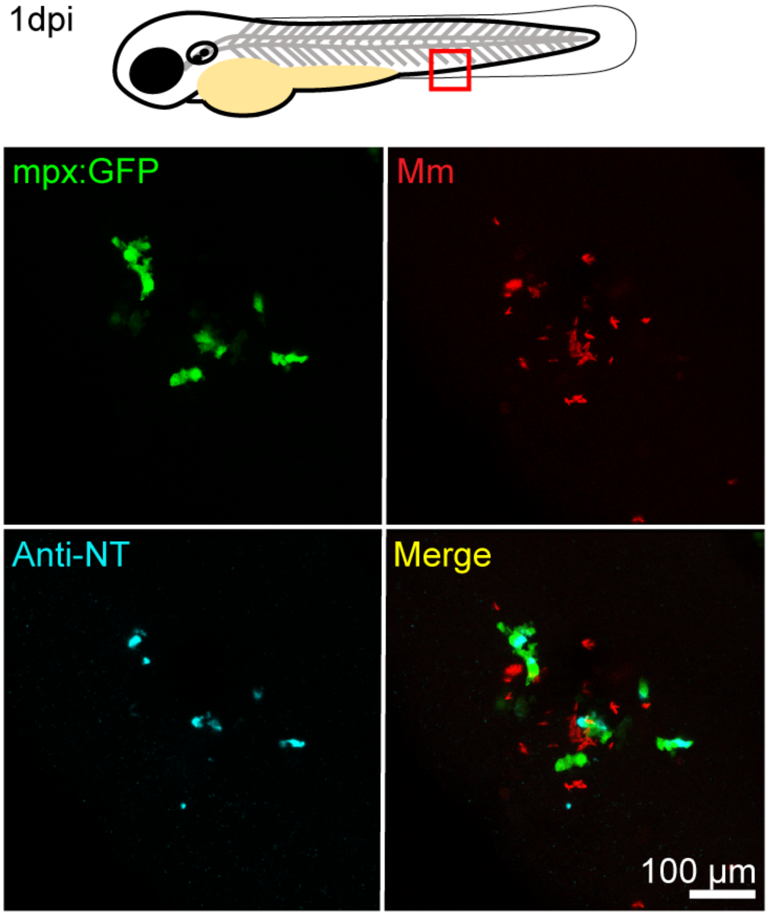

Figure S4. Anti-nitrotyrosine signal is predominantly found in neutrophils after Mm infection.

(A) Example fluorescence confocal z-stacks of the caudal vein region of *Tg(mpx:GFP)i114* embryos (green neutrophils) stained with Alexa-633 labelled anti-nitrotyrosine antibody (cyan), imaged at 1dpi in the presence of Mm mCherry infection (red).
