# Supplementary material for: Hif-1α–Induced Expression of Il-1β Protects against Mycobacterial Infection in Zebrafish
Source: J Immunol. 2018 Dec 14;202(2):494–502. doi: 10.4049/jimmunol.1801139 (PMC6321843; doi:10.4049/jimmunol.1801139)
Supplement: Data Supplement [file JI_1801139.zip › JI_1801139_Supplemental_Material_1.pdf]

Supplemental Movie 1. *Il-1β:GFP* expression in activated immune cells after Mm infection.

Fluorescent confocal videotimelapse of *il-1β:GFP* in immune cells containing Mm infection (*il-1β:GFP* in green and Mm mCrimson in red).
